# Oral calcium supplementation versus placebo in mitigating citrate reactions during apheresis: an open-label randomized control trial

**DOI:** 10.1016/j.htct.2024.06.010

**Published:** 2024-09-20

**Authors:** Masaya Abe, Keiko Fujii, Nobuharu Fujii, Toshiharu Mitsuhashi, Takuya Fukumi, Yuichi Sumii, Maiko Kimura, Tomohiro Urata, Takumi Kondo, Fumio Otsuka, Yoshinobu Maeda

**Affiliations:** a Department of Hematology, Oncology and Respiratory Medicine, Okayama University Graduate School of Medicine, Dentistry and Pharmaceutical Sciences, Okayama, Japan; b Division of Transfusion, Okayama University Hospital, Okayama, Japan; c Division of Clinical Laboratory, Okayama University Hospital, Okayama, Japan; d Department of Hematology and Oncology, Okayama University Hospital, Okayama, Japan; e Center for Innovative Clinical Medicine, Okayama University Hospital, Okayama, Japan; f Department of General Medicine, Dentistry and Pharmaceutical Sciences, Okayama University Graduate School of Medicine, Okayama, Japan

**Keywords:** Allogeneic peripheral blood progenitor cell collection, Citrate-related adverse effect, Oral calcium drink, Ionized calcium

## Abstract

**Background:**

Citrate-related hypocalcemia is the most common adverse event linked with peripheral blood progenitor cell apheresis. A previous retrospective study highlighted the prophylactic effectiveness of oral calcium drinks before apheresis, supplemented with intravenous calcium gluconate. Consequently, this study is a randomized controlled trial comparing oral calcium with placebo drinks

**Study Design and Methods:**

Healthy donors were randomized to receive either oral calcium (Cohort A) or placebo (Cohort B) drinks. If symptoms emerged, all donors were given calcium drinks to counteract hypocalcemia. The primary endpoint centered on the incidence of Grade 1 or higher citrate-related symptoms. Analyses were performed using the crude model and doubly robust estimation.

**Results:**

Forty-two healthy donors participated from January 2021 to July 2022. Case distribution (Cohort A: Cohort B) stood at 3:7 (Grade 1), 2:2 (Grade 2), and 1:0 (Grade 3); no Grade 4 cases were identified. There was no statistical significance in the incidence of Grade 1 or higher and Grade 3 citrate-related symptoms.

**Discussion:**

The cumulative incidence of citrate-related side effects was less pronounced than in the previous research. This could stem from absence of blinding, and the decision to administer calcium drinks to the untreated group upon symptom detection. Although preemptive oral calcium intake before peripheral blood progenitor cell apheresis is not wholly effective, providing calcium-rich beverages to symptomatic donors may stave off symptom intensification.

## Introduction

Peripheral blood progenitor cells (PBPCs) serve as a primary graft source for both allogeneic and autologous hematopoietic stem cell transplantation^1^. The harvesting of PBPCs necessitates apheresis, which employs citric acid as an anticoagulant. When introduced, citric acid binds to ionized calcium, leading to decreased blood levels of this essential ion. This reduction can result in citrate-induced hypocalcemia, commonly referred to as citrate-related reactions. These reactions represent the predominant adverse event linked to PBPC apheresis, with manifestations varying from mild paresthesia of the limbs and mouth to more severe outcomes like convulsions and cardiac arrhythmias.^2^

Platelet apheresis is a well-known form of apheresis performed in healthy donors. Active research is being conducted to prevent adverse events during platelet apheresis because the platelets are collected from volunteer donors.^3^, ^4^, ^5^ PBPC apheresis is longer than platelet apheresis, with PBPC donors receiving more citric acid and experiencing more adverse events. Therefore, PBPC apheresis generally involves the prophylactic administration of intravenous calcium gluconate, which is not used in platelet apheresis.^6^ Fatal hypocalcemia has been avoided by (i) prophylactic administration of intravenous calcium gluconate if symptoms appear, (ii) increasing or temporarily administering a bolus of calcium gluconate, or (iii) temporarily suspending apheresis or reducing the blood flow rate.^6^^,^^7^ Although studies have been conducted to prevent adverse events during PBPC apheresis,^7^, ^8^, ^9^ the prophylactic effect has been insufficient. Mild symptoms such as numbness of the limbs and mouth are common. There have been no reports of preventive methods to completely suppress citrate-related reactions, including in mild cases.

The authors previously analyzed 80 healthy donors who underwent PBPC apheresis using an inverse probability weighted (IPW) regression adjustment (RA) method. Adding oral calcium drinks to intravenous calcium gluconate reduced the frequency of citrate-related reactions by 39.2 % point (p.p.).^10^ Calcium-based drinks are conventionally consumed; hence, proving the prophylactic effect of calcium drink consumption on the onset of citrate-related symptoms will be very useful. Therefore, this prospective randomized open-label comparative trial was designed.

## Materials and methods

### Trial design

This open-label, two-group, randomized study enrolled donors aged 15–65 years if they were eligible for donor screening for PBPC apheresis. All the donors received standard intravenous calcium gluconate prophylaxis. Donors were randomly assigned in a 1:1 ratio to take oral calcium (Cohort A) or placebo (Cohort B) drinks before starting apheresis. Cohort B was allowed to take oral calcium drinks if symptoms occurred. The adjustment factor for minimization was being female. This prospective analysis was conducted in accordance with the Declaration of Helsinki and approved by the Okayama University Certified Review Board. Written informed consent was obtained from all the participants.

### Apheresis procedure of peripheral blood progenitor cells harvesting

PBPCs were harvested using the two-needle method with a continuous cell separator, Spectra Optia (Terumo BCT, Inc., Lakewood, CO, USA), and harvesting was completed using the continuous mononuclear cell collection protocol. The target cell count, ranging from 150 to 300 mL/kg, was set to be collected in one day with as much processed blood volume as possible. Cases involving PBPC collection on two consecutive days were included in the study only on the first day. Acid citrate-dextrose A (ACD-A; Terumo Corporation, Tokyo, Japan) was used as an anticoagulant. The blood-to-ACD-A ratio was initially set at 12:1, with changes to 13:1 in some cases. The packing factor was set to 4.0.

### Calcium supplementation

All donors received continuous intravenous administration of calcium gluconate hydrate (Calcicol, Nichi-Iko Pharmaceutical Co., Ltd., Toyama, Japan; calcium gluconate 8.5 % [wt/vol]; 0.39 mEq/mL of calcium; pH 6.0–8.2) at 22 mL/h during apheresis. Donors in Cohort A were administered 250 mL of calcium drinks (Calgen mini: Calgen Pharmaceuticals, Inc., Osaka, Japan) containing 66.3 kcal of energy, 16.3 g of carbohydrates, 6.8 mg of sodium, 19.8 mg of potassium, 130 mg of calcium, 11.5 mg of phosphorus, and 0.01 mg of vitamin B2 per 125 mL. Conversely, donors in Cohort B were administered 250 mL of placebo drinks (Calpis water: Calpis Co., Ltd., Tokyo, Japan) containing 45 kcal of energy, 11 g of carbohydrates, about 10 mg of potassium, and less than 10 mg of calcium per 100 mL. Constant monitoring for hypocalcemia symptoms was performed during PBPC apheresis. Additionally, constant monitoring of heart rate for arrhythmia was performed using electrocardiography devices. If mild paresthesia developed, all donors in Cohort B, except those who refused to drink, consumed a calcium drink. If the symptoms persisted, the infusion rate of calcium gluconate hydrate was increased to 25–30 mL/h.

### Evaluation of hypocalcemic symptoms

Experienced doctors assessed hypocalcemic symptoms such as paresthesia, cramping, and nausea. Then the symptoms were graded using a system based on a study by Bolan et al.^11^ The doctor assigned a grade of (i) ‘0’ if no symptoms were noted; (ii) ‘1’ if symptoms were barely noticeable (mild paresthesia); (iii) ‘2’ if symptoms were irritating (moderate paresthesia); (iv) ‘3’ if symptoms were uncomfortable (severe paresthesia, nausea, nervousness, and anxiety); and (v) ‘4’ if symptoms were unbearable (arrhythmia, tetany, and seizures).

### Detection of ionized calcium

One mL of whole blood was collected from the upstream venous line of the calcium gluconate line before commencing PBPC apheresis, at intervals of 0, 15, 30, 45, 60, and 90 min, and at the end of apheresis. Ionized calcium levels in whole blood were measured with added heparin using Stat Profile pHOx (Nova Biomedical K.K., Tokyo, Japan) within 10 min of collection.

### Outcomes

The primary endpoint was the incidence of Grade 1 or higher citrate-related reactions. Secondary endpoints included the incidence of Grade 3 or higher citrate-related reactions, changes in ionized calcium levels in the early period (15 and 30 min) after commencing apheresis, and safety. Changes in ionized calcium levels were calculated using the following mathematical formula:

Change in ionized calcium (%) = [(ionized calcium level at start) – (ionized calcium level at each timepoint)] / (ionized calcium level at start) × 100.

### Statistical analysis

The sample size of 42 was calculated as follows: assuming a 78 % probability of Grade 1 or higher citrate-related reactions in the placebo group and a 40 % probability in the calcium drink group based on the authors’ previous report,^10^ an alpha error of 0.05 (two-sided) and a beta error of 0.2, the required number of participants was calculated as 40. Assuming a 5 % dropout, 42 (21 per group) participants were selected. Stata (Stata Corporation, version 16.1, College Station, TX, USA) and GraphPad Prism 8 software (GraphPad Inc., San Diego, CA, USA) were used for all statistical analyses. All *p*-values were two-sided, and those less than 0.05 were considered statistically significant. Means and standard deviations are used to describe normally distributed continuous variables. In comparison, medians and interquartile ranges are used to describe non-normally distributed continuous variables. Categorical variables are described as frequencies and percentages.

A time series of ionized calcium levels was drawn for each cohort and symptom, and the differences at each timepoint were tested using Welch's *t*-test. The differences in covariates between Cohorts A and B are represented as standardized mean differences (SMDs) and variance ratios (VRs). The absolute SMD values of less than 0.25 and VRs in a range of 0.5–2.0 were considered balanced.^12^^,^^13^ As this study was randomized; the covariates should be balanced in Cohorts A and B. Therefore, the outcomes between groups were compared in the analysis without adjusting for covariates (crude model). However, if they were found to be unbalanced, the inverse probability weighted regression adjustment (IPWRA) model was used to address potential confounding factors, chosen based on consensus among the investigators. Using this model introduced double robustness into the analyses.^14^, ^15^, ^16^ The treatment model (IPW model) predicted the probability of drinking calcium drinks. In comparison, the outcome model (regression adjustment model) used a statistical model to predict the outcome. The variables used for these predictions in both models were age, sex, height, weight, total blood volume, maximum blood flow, ACD-A injection rate, pre-apheresis CD34^+^ count, total processing time, the total amount of ACD-A, and total processing volume. Using a weighted outcome model, the average treatment effect estimate was calculated as the difference between the average predicted outcomes.

## Results

### Donor characteristics and results of peripheral blood progenitor cell apheresis

Between January 2021 and July 2022, 42 healthy donors (21 each in Cohorts A and B) underwent randomization. Before IPW matching, the donor characteristics were greater than 0.25 in SMDs, including age, height, weight, maximum blood flow, ACD-A injection rate, total processing time, total amount of ACD-A, and total processing volume. After using IPW matching, they were greater than 0.25 in SMDs in the total amount of ACD-A and total processing volume (Table 1).

**Table 1:** Clinical donor characteristics and detailed results of the apheresis, standardized mean difference, and variance ratio before and after inverse probability weighting regression adjustment

	Before IPW				After IPW			
	Cohort A (Calcium drinks)	Cohort B (Placebo drinks)	SMD	VR	Cohort A (Calcium drinks)	Cohort B (Placebo drinks)	SMD	VR
Clinical characteristic								
Number/weighted number	21	21			20.7	21.4		
Female^a^	7 (33.3 %)	8 (38.1 %)	0.097	0.942	6 (29.7 %)	7 (34.9 %)	0.109	0.919
Age	32.9 ± 13.2	39.0 ± 12.6	−0.472	1.090	35.6 ± 15.0	37.4 ± 12.5	−0.135	1.427
Height (cm)	168.1 ± 10.3	165.3 ± 10.4	0.277	0.971	167.2 ± 9.5	166.5 ± 10.6	0.066	0.806
Weight (kg)	63.4 ± 12.6	67.0 ± 14.6	−0.262	0.738	65.1 ± 12.0	66.8 ± 14.0	−0.133	0.732
TBV (mL)	4270.1 ± 864.8	4278.0 ± 892.9	-0.009	0.938	4305.9 ± 808.0	4322.0 ± 890.4	−0.019	0.824
Detailed results of the apheresis								
Maximum blood flow (ml/min)	67.8 ± 9.9	70.5 ± 10.4	−0.267	0.913	69.2 ± 10.5	69.5 ± 10.8	−0.035	0.944
ACD-A injection rate	1.3 ± 0.1	1.3 ± 0.1	−0.285	0.953	1.3 ± 0.1	1.3 ± 0.1	−0.018	0.941
Pre-apheresis CD34^+^ count (/μl)	50.0 ± 31.9	46.2 ± 30.0	0.120	1.126	49.3 ± 29.3	48.5 ± 27.9	0.029	1.109
Total processing time (min)	209.4 ± 48.7	240.0 ± 64.4	−0.536	0.573	212.8 ± 48.0	225.3 ± 59.0	−0.232	0.662
Total amount of ACD-A (mL)	977.0 ± 273.80	1164.9 ± 345.6	−0.603	0.627	1010.2 ± 263.9	1082.8 ± 301.7	−0.256	0.765
Total processing volume (mL)	11379.7 ± 3164.9	13710.3 ± 4168.4	−0.630	0.576	11749.8 ± 3071.4	12644.1 ± 3701.4	−0.263	0.687
Mean of absolute value of SMD/VR			0.323	0.859			0.118	0.892

Values represent means ± standard deviation.

ACD-A: Acid citrate-dextrose A; IPW: Inverse probability weighting; TBV: Total blood volume; SMD: Standardize mean difference; VR: Variance ratio.

a Categorical variables, display frequencies and percentages.

### Citrate-related symptoms and safety

Regarding citrate-related reactions observed in the 21 donors in Cohort A, 15 donors were asymptomatic (Grade 0), three developed Grade 1 symptoms, two developed Grade 2 symptoms, one developed Grade 3 symptoms, and none developed Grade 4 symptoms. Of the 21 donors in Cohort B, 12 were asymptomatic (Grade 0), seven developed Grade 1 symptoms, two developed Grade 2 symptoms, and none developed Grade 3 or Grade 4 symptoms (Table 2). Grade 1 or higher symptoms were 14.3 p.p. less likely in Cohort A than in Cohort B in the crude model and 9.8 p.p. less likely in the doubly robust estimation. Grade 3 symptoms were 4.8 p.p. more likely in Cohort A in the crude model and 10.5 p.p. more likely in the doubly robust estimation; however, this difference was insignificant (Table 3). Grade 4 symptoms were not observed in either group. Serious adverse events, including citrate-related reactions that required apheresis interruption or discontinuation, were not observed.

**Table 2:** Number of citrate-related reactions in each cohort

Citrate-related reactions	Cohort A (Calcium drinks)	Cohort B (Placebo drinks)
	n = 21	n = 21
Grade 0	15	12
Grade 1	3	7
Grade 2	2	2
Grade 3	1	0
Grade 4	0	0

**Table 3:** Comparison of citrate-related reactions by grade in each cohort

			Crude model		Doubly robust model	
Citrate-related reactions	Cohort A (Calcium drinks)	Cohort B (Placebo drinks)	Risk difference	*p*-value	ATE	*p*-value
	n = 21	n = 21	(95 % CI)		(95 % CI)	
Grade 1 or higher	6	9	−0.143	0.329	−0.098	0.455
Incidence risk	0.286	0.429	(−0.429 to 0.144)		(−0.354 to 0.159)	
Grade 3	1	0	0.048	0.306	0.105	0.130
Incidence risk	0.048	0	(−0.043 to 0.139)		(−0.031 to 0.242)	

CI: Confidence interval; ATE: Average treatment effect

### Transition and changes in ionized calcium levels

**Figure 1:**
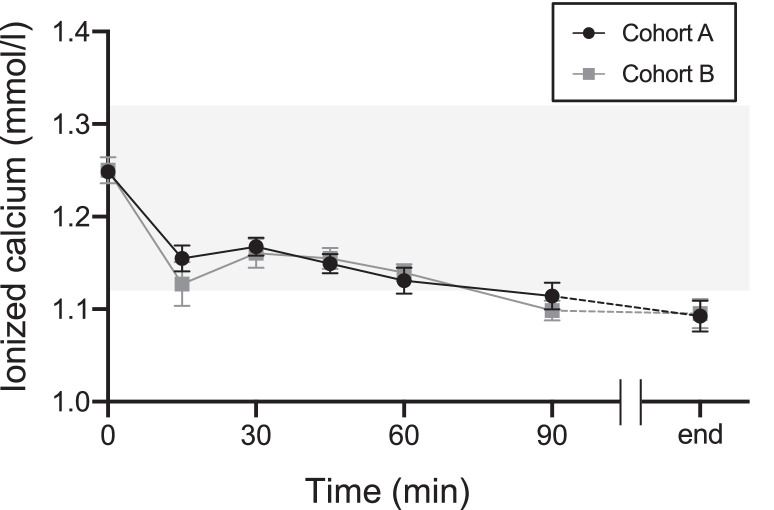
The transition of the level of ionized calcium in Cohorts A (calcium drinks) and B (placebo drinks) The black line indicates Cohort A. The gray line indicates Cohort B. Tinted area indicates reference ranges of ionized calcium (1.12–1.32 mmol/L). Values represent means ± standard error of the mean.

Figure 1 shows the changes in ionized calcium levels at 0, 15, 30, 45, 60, and 90 min after the start and at the end of apheresis. Ionized calcium levels decreased at 15 min compared with those at 0 min in both cohorts. They increased transiently at 30 min, then slowly declined until the apheresis ended. Changes in ionized calcium levels were calculated in the early period (at 15 and 30 min) and the maximum change during apheresis was calculated. Due to missing values, the number of donors for the early analysis period was 40. The changes at 15 min and 30 min and the maximum change during apheresis were smaller in Cohort A than in Cohort B; however, this difference was insignificant (Table 4).

**Table 4:** Changes in ionized calcium in the early period after the start of apheresis and maximum changes in the apheresis

After starting apheresis			Crude model		Doubly robust model	
Change in ionized calcium (%)	Cohort A (Calcium drinks)	Cohort B (Placebo drinks)	Change difference	*p*-value	ATE	*p*-value
			(95 % CI)		(95 % CI)	
n	20	20	−2.389	0.182	−2.252	0.197
15 min	7.437 ± 2.176	9.825 ± 2.590	(−5.897 to 1.119)		(−5.677 to 1.173)	
n	19	21	−0.724	0.334	−0.348	0.518
30 min	6.452 ± 1.521	7.176 ± 1.596	(−2.192 to 0.744)		(−1.406 to 0.709)	
n	21	21	−1.063	0.545	2.272	0.302
Maximum	14.936 ± 2.567	15.999 ± 2.227	(−4.507 to 2.380)		(−2.041 to 6.586)	

Values represent means ± standard deviation

CI: Confidence interval; ATE: Average treatment effect

### Transition of ionized calcium between donors with and without symptoms in each group

**Figure 2:**
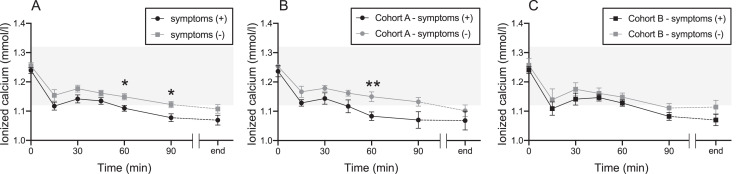
The transition of the level of ionized calcium between donors with and without symptoms in each cohort Cohort A took oral calcium drinks, and Cohort B took placebo drinks. (A) Combined for both Cohorts A and B. (B) Cohort A alone. (C) Cohort B alone. The black line indicates the group with symptoms. The gray line indicates the group without symptoms. Tinted area indicates reference ranges of ionized calcium (1.12–1.32 mmol/L). Values represent means ± standard error of the mean. *p-value < 0.05, **p-value < 0.01.

No difference in changes in ionized calcium levels was observed between Cohorts A and B. However, when all donors with and without symptoms were retrospectively compared, donors with symptoms showed decreased ionized calcium levels at 60 and 90 min (Figure 2A). Compared to those with and without symptoms in Cohort A, the ionized calcium levels of donors with symptoms declined at 60 min (Figure 2B). In Cohort B, there was no difference in ionized calcium levels between symptomatic and asymptomatic donors (Figure 2C).

## Discussion

The results indicate that the incidence of citrate-related reactions of Grade 1 or higher showed no significant variance between Cohorts A and B. Equally, there were no discernible differences in ionized calcium levels during the initial post-apheresis stages. A closer examination of the apheresis results between Cohorts A and B revealed a few distinctions. Notably, Cohort B had a protracted processing time, which was found to have a direct positive correlation with the onset of citrate-related reactions. This extended processing time was also strongly associated with allocation to Cohort B. It is plausible that the analysis with variables, including this processing time, might have influenced the risk of onset of citrate-related reactions in Cohort A.

**Figure 3:**
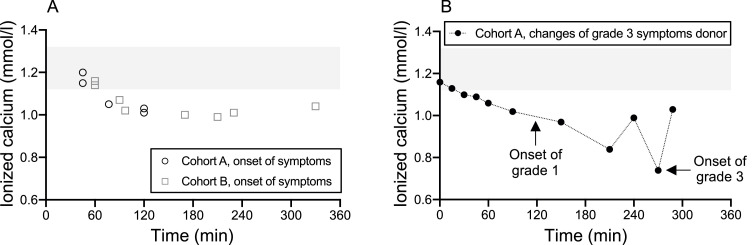
The onset time and the level of ionized calcium. Cohort A took calcium drinks and Cohort B took placebo drinks before apheresis. (A) Cohort A is represented by the open circles representing donors with Grade 1 or 2 hypocalcemic symptoms. Cohort B is represented by the open squares and indicates the onset of symptoms of any grade. (B) The donor in Cohort A with Grade 3 hypocalcemic symptoms is represented with the closed circles with the black line representing the change of ionized calcium. Arrows supplement Grade 1 and Grade 3 onset times. Tinted area indicates reference ranges of ionized calcium (1.12–1.32 mmol/L).

A study by Kishimoto et al. highlighted that five autologous PBPC apheresis donors, upon consuming isotonic sports drinks enriched with calcium at the onset of symptoms, witnessed a significant rise in ionized calcium levels within a short 2- to 5-min interval. This elevated level remained acceptable for a subsequent 31–55 min.^7^ Our data, as depicted in Table 5 and Figure 3A, show the onset times of citrate-related reactions alongside ionized calcium readings. Donors from both cohorts consumed their respective drinks roughly 15.67 ± 5.91 and 13.90 ± 3.77 minutes before apheresis began. A majority of the donors exhibited symptom onset times that exceeded 60 min. This suggests that the consumption of just one calcium drink prior to apheresis might be insufficient to counteract the decline in ionized calcium during prolonged apheresis sessions. Interestingly, within our study parameters, certain donors showed symptoms within the first 60 min, yet retained standard ionized calcium levels at the onset of symptoms. Earlier research posits that ionized calcium does not always diminish entirely upon the emergence of symptoms. Rapid declines in these levels are potentially symptomatic triggers.^7^^,^^17^ Figure 3B illustrates the fluctuations in ionized calcium levels juxtaposed against the onset times for Grade 1 and Grade 3 symptoms in a Cohort A donor who displayed indications of Grade 3 hypocalcemia. A precipitous decline in ionized calcium was observed preceding the emergence of Grade 3 symptoms, possibly intensifying the symptoms.

**Table 5:** Onset time of citrate-related reactions (n = 15)

Symptoms onset time (min)	≤ 60	> 60 and ≤ 90	> 90 and ≤ 120	> 120 and ≤ 150	> 150 and ≤ 180	> 180
n	4	2	4	1	1	3
%	26.67	13.33	26.67	6.67	6.67	20

In our observations, only one donor manifested Grade 3 symptoms, and notably, none escalated to Grade 4. Drawing from our earlier retrospective analysis, of the 23 donors who abstained from pre-apheresis calcium drinks, four had Grade 3 or higher symptoms. In stark contrast, none of the 57 donors who drank calcium drinks experienced such symptoms.^10^ Keeping in mind the ethical considerations and based on the precedent of a relatively elevated symptom onset rate and the onset of Grade 3 or higher symptoms in previous studies, the placebo group in this trial was also administered calcium drinks upon symptom emergence. Consequently, the risk metrics in this study proved more favorable than earlier projections.^10^ This diminished incidence of severe symptoms might be attributed to the timely provision of calcium beverages upon symptom onset. Though the proficiency of the research team in handling symptom emergence can sway results, the early intervention with calcium drinks may be pivotal in curbing symptom severity. Given the utility and timely deployment of these calcium drinks, one must approach the extrapolation of severe case incidence risk in Cohort B to the broader populace with prudence.

Potential limitations of this study include the possibility that the actual incidence and severity perceptions of citrate-related reactions may have been influenced by the absence of blinding. Another point of contention is the potential of an unidentified confounding bias skewing the crude model and doubly robust estimate, considering the significant disparity in background characteristics across the cohorts.

Our open-label, randomized controlled study did not conclusively establish the advantage of preemptively administering oral calcium drinks before apheresis over the conventional intravenous calcium. As such, innovative strategies might be imperative to thwart the immediate drop in post-apheresis ionized calcium and its gradual decline after the 60-min mark. Moving forward, we are contemplating another randomized controlled trial, utilizing a refined pharmaceutical formula to ensure optimal calcium consumption. Despite these exploratory stages, our current study fortifies the foundation for enhancing the safety protocols in all apheresis procedures.

## The sources of support in the form of grants, equipment, or drugs

There were no grants given for this study.

## CRediT authorship contribution statement

Masaya Abe: Writing - original draft. Keiko Fujii: Conceptualization, Designed the study, Methodology, Writing - original draft. Nobuharu Fujii: Conceptualization, Methodology, Writing - review & editing. Toshiharu Mitsuhashi: Validation, Formal analysis. Takuya Fukumi: Investigation. Yuichi Sumii: Investigation. Maiko Kimura: Investigation. Tomohiro Urata: Investigation. Takumi Kondo: Investigation. Fumio Otsuka: Supervision. Yoshinobu Maeda: Supervision.

## Conflicts of interest

The authors report no declarations of interest.
